# Systematic review of digital self‐management interventions in dementia care: Exploring the impact of wearable technology, smart homes, and mobile applications on patient outcomes

**DOI:** 10.1002/alz.088900

**Published:** 2025-01-09

**Authors:** Gary Cornelius

**Affiliations:** ^1^ University of Strathclyde, Glasgow, Scotland United Kingdom

## Abstract

**Background:**

The increasing significance of self‐management in dementia care arises from earlier diagnosis, improved understanding of patient‐modifiable factors, and advancements in treatments. The growing acceptance of patient care planning, especially self‐management, is further supported by health professionals and public health initiatives aimed at extending healthspan.

**Method:**

This systematic review evaluates the efficacy of self‐management tools in enhancing the quality of life for adults with dementia and mitigating undesirable behaviours associated with the condition. A comprehensive search spanning ten years across nine databases was conducted, leading to the screening of over eight hundred articles. A highly relevant collection of studies, focusing on adults with dementia’s interaction with digital technologies, was selected. These studies were analysed using the Mixed Methods Appraisal Tool (MMAT) and a custom‐designed characterization table to address specific research questions.

**Result:**

The review highlights that self‐management approaches such as aiding functional independence, wellbeing support, engagement, monitoring, and detecting abnormal behaviour are effective. The studies utilized various assessment methods including multimodal sensing, caregiver feedback, and machine‐based prompting. Effective use of smart home sensors, health watches, wearable technologies, cameras, Internet of Things (IoT) networks, robots, and other devices was also noted.

**Conclusion:**

Technologies for self‐management notably enhance patient well‐being, with effective strategies including monitoring for abnormalities, socially assistive robots, machine prompting, sensory interventions, and cognitive support. Future research could improve by aligning interventions with individualized activities that promote well‐being, employing standardized outcome measures, and controlling for external factors such as known patient disease‐modifying factors.

